# Design of a Dual-Band Broadband Antenna Based on Structure Reuse

**DOI:** 10.3390/mi17020257

**Published:** 2026-02-16

**Authors:** Huiru Zhang, Junwen Tang, Zhongjun Yu

**Affiliations:** 1 School of Electronic, Electrical and Communication Engineering, University of Chinese Academy of Sciences, Beijing 101408, China; zhanghuiru21@mails.ucas.ac.cn; 2 Aerospace Information Research Institute, Chinese Academy of Sciences, Beijing 100190, China; tangjunwen@aircas.ac.cn

**Keywords:** dual-band antenna, structure reuse, slot antenna, microstrip patch

## Abstract

In this paper, a novel dual-band broadband antenna based on structure reuse is proposed. The proposed antenna integrates a slot antenna with a microstrip antenna to achieve dual-band performance. The slot antenna innovatively serves as both a radiating element and a feeding structure for the microstrip antenna, realizing structure reuse and significantly reducing structural complexity. To enhance the dual-band bandwidth, four symmetrically arranged parasitic strips are introduced, effectively extending the low-frequency bandwidth. Additionally, the high-frequency bandwidth is further improved by the introduction of a U-shaped slot. To analyze its working principle, the characteristics of the current and electric field distributions at each resonant point are given. The measured results indicate that in the low-frequency band, the proposed antenna achieves a relative bandwidth of 22.1% and a peak gain of 6.5 dBi. In the high-frequency band, it realizes a relative bandwidth of 13.6% and a peak gain of 4.6 dBi.

## 1. Introduction

With the significant advancement of wireless communication technologies, applications such as cellular base stations and satellite communications have increasing requirements for antenna performance. At present, the focus of satellite communication research has gradually shifted to the K and Ka frequency bands. Compared to lower frequency bands, these two bands can provide richer spectral resources, thereby enabling greater system capacity and more miniaturized terminal devices. However, achieving high performance across dual frequency bands within a constrained physical space has become a critical challenge in antenna design, making research on dual-band antennas a topic of significant interest [1,2,3,4,5]. Currently, two typical methods are employed to achieve dual-band antennas. The first approach implements multi-band antennas through the spatial arrangement of multiple independent antenna elements [6,7]. However, this approach faces multiple technical challenges. On one hand, the complexity of the feed network inevitably increases as each frequency band requires a separate matching network. On the other hand, mutual coupling between bands can severely degrade radiation performance.

The second approach achieves dual-band operation through a single antenna element. For example, one typical method involves utilizing different operating modes of the antenna, such as the fundamental mode and higher-order modes corresponding to distinct frequency bands [8,9,10,11,12,13,14]. However, this method has inherent limitations, including distinct radiation patterns across operating modes, a narrow operational bandwidth, and resonant frequencies constrained by the frequency ratio between two modes. In ref. [14], four L-shaped open-circuit stubs are added at the non-radiating boundaries to excite two resonant modes. However, the measured results indicate that the achieved bandwidths are only 3.1% and 9.2%, demonstrating clearly limited overall bandwidth performance. The other method achieves dual-band operation by utilizing multiple resonant structures [15,16,17,18,19,20,21,22,23], such as stacked patches [15,16,17] and parasitic structures [18,19,20,21,22,23]. In ref. [16], a dual-band antenna is realized by employing three stacked patches, achieving bandwidths of 3.6% and 9.1%. Although dual-band characteristics are realized, this design not only increases the antenna profile but also results in relatively narrow bandwidths for both bands.

In addition, the method of etching slots on patches is also commonly used to achieve dual-band performance [24,25]. In ref. [25], a dual-band antenna is realized by utilizing the fundamental mode of a microstrip patch and etching trapezoidal slots on the patch. However, the achieved bandwidths are only 4.8% and 6.09%, which are insufficient to meet the requirements for wideband applications. Although existing designs have achieved dual-band performance, they still face many challenges such as narrow bandwidth, large spatial footprint, and high profile. Therefore, it remains challenging and practically significant to achieve an antenna with dual-band operation, wide bandwidth, and a simple structure.

In this work, a novel dual-band broadband antenna design based on structure reuse is proposed. The design integrates a slot antenna and a microstrip antenna to achieve dual-band performance. The slot antenna innovatively serves as both a radiating structure and a feeding structure for the microstrip antenna, thereby successfully achieving structure reuse and significantly reducing the overall complexity. Furthermore, parasitic strips and a U-shaped slot are added to introduce additional resonant points, thereby further enhancing the broadband performance of each frequency band. The current and electric field distributions at each resonant point are studied in detail to analyze its working mechanism. The proposed antenna achieves bandwidths of 17.9–22.35 GHz and 27.24–31.2 GHz, demonstrating excellent dual-band broadband performance in both bands. The design evolution, working principle, and experimental results are systematically described in the following section.

## 2. Antenna Configuration and Evolution

### 2.1. Antenna Configuration

**Figure 1:**
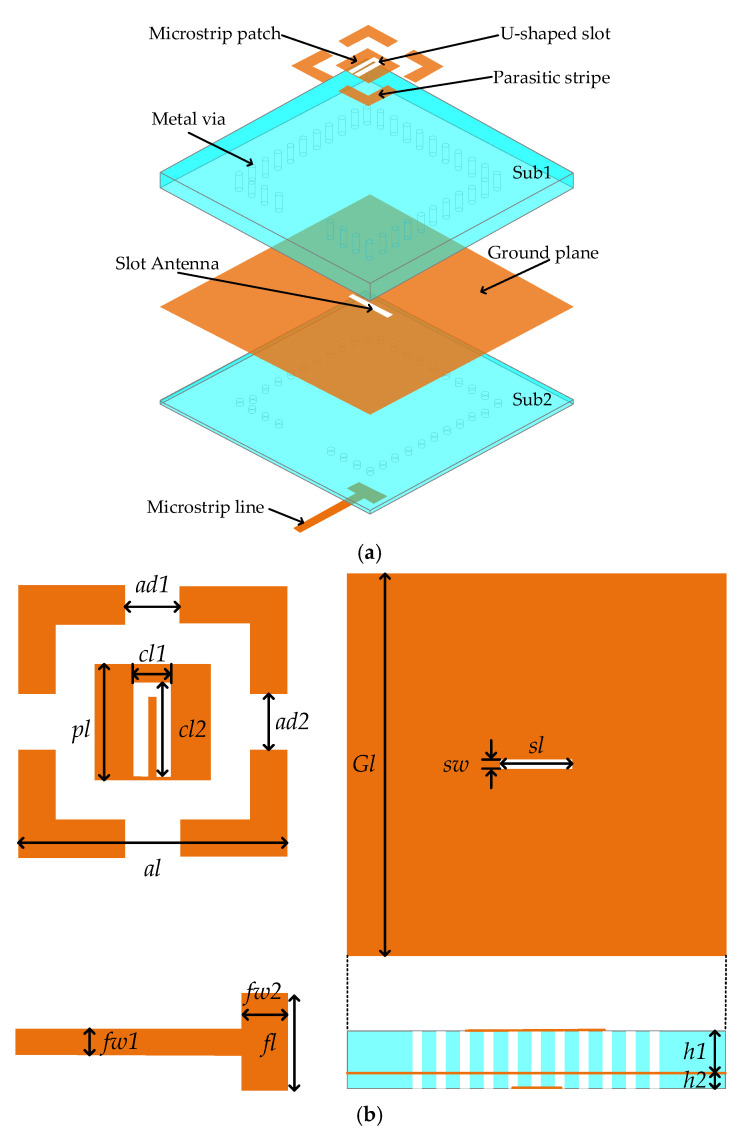
Structural configuration of the designed antenna. (**a**) Three-dimensional perspective; (**b**) Detailed perspective.

This work presents a dual-band broadband antenna based on structure reuse, as illustrated in Figure 1. The main dielectric layer is composed of Rogers 4350 (ε_r_ = 3.66, tanδ = 0.004) with thicknesses of 0.422 mm and 0.254 mm, while the prepreg layer employs Rogers 4450 (ε_r_ = 3.52, tanδ = 0.004) with a thickness of 0.101 mm to ensure both electrical performance and mechanical stability. The structure comprises a slot antenna, a microstrip patch, a U-shaped slot, parasitic strips, metal vias, a T-shaped microstrip line, and a ground plane.

To achieve dual-band performance, this design combines a slot antenna with a microstrip patch. Based on structure reuse, this design utilizes a single structure to achieve dual functionalities. The slot antenna innovatively integrates both radiating and feeding functions, thereby effectively enhancing the antenna’s compactness and performance. To further broaden the operating bandwidth of both frequency bands, parasitic strips and a U-shaped slot are introduced. The proposed antenna is fed by a T-shaped microstrip line printed on the bottom layer, with its T-shaped structure designed to optimize the antenna’s impedance matching. Meanwhile, the metal vias form a metal wall, with its core function being to maintain the effective ground size of the antenna unchanged. This ensures that the antenna performance remains unchanged when the physical ground plane is expanded due to assembly requirements. The dimensions of the designed structure are summarized in Table 1.

### 2.2. Antenna Evolution

**Figure 2:**
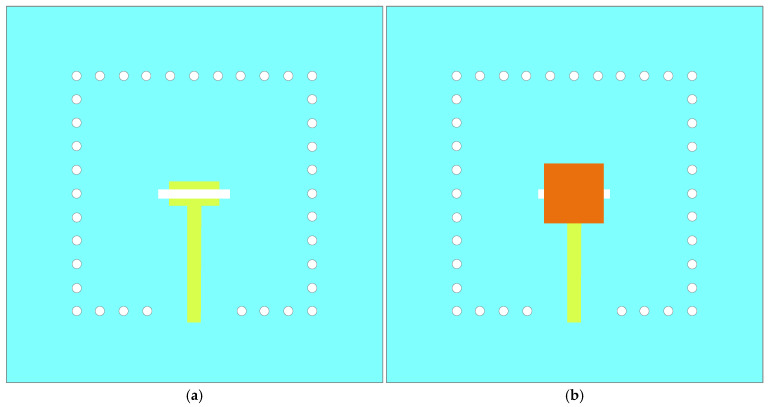

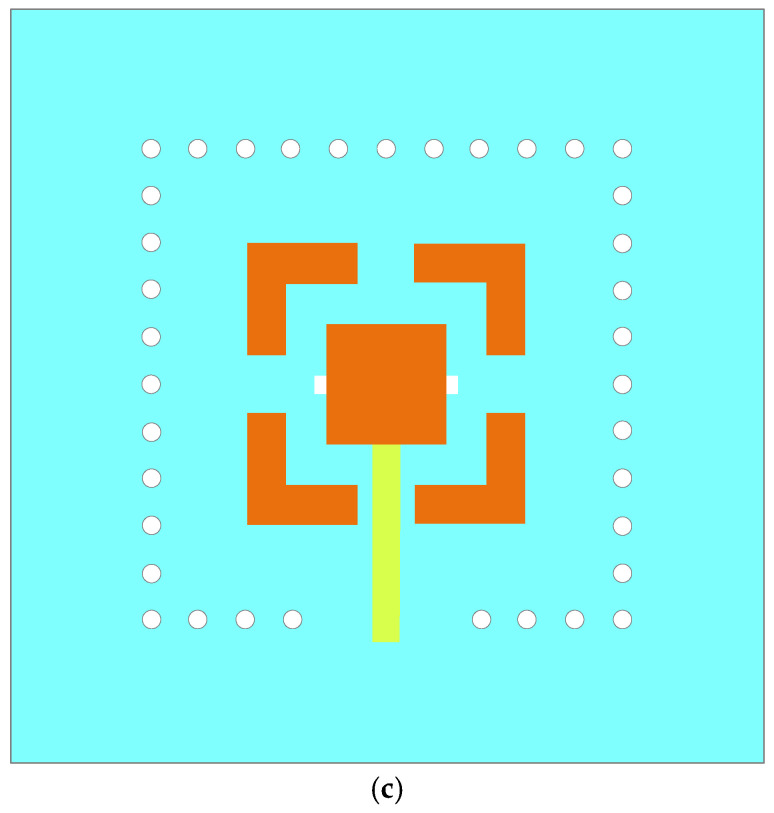
Evolution of the design geometry. (**a**) Ant. A; (**b**) Ant. B; (**c**) Ant. C.

**Figure 3:**
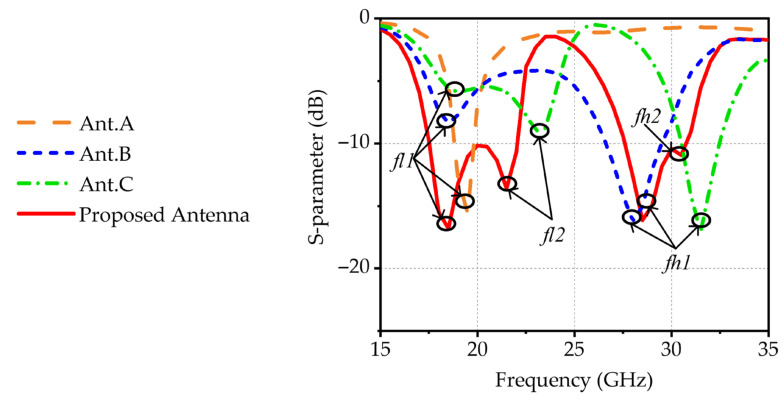
Comparison of reflection coefficients between the reference and proposed antennas.

To better clarify the operating principle of the novel design, its performance is systematically compared with three reference designs (Ant. A, Ant. B, and Ant. C) in Figure 2. Ant. A is a microstrip-fed slot antenna, in which energy is coupled from the microstrip line to the slot for radiation, with its structure depicted in Figure 2a. A resonant point, labeled as *fl1*, is obtained in the low-frequency band, as depicted in Figure 3. To achieve dual-band characteristics, a novel dual-band antenna based on structure reuse is proposed. As illustrated in Figure 2b, Ant. B introduces a microstrip patch above the slot antenna. In this configuration, the slot antenna innovatively serves functions as both a radiating structure and a feeding structure for the patch, enabling structure reuse and reducing overall complexity. The energy is coupled from the slot antenna to the microstrip patch, exciting its fundamental mode and achieving radiation. The corresponding resonant frequency is determined by the dimensions of the patch. The results in Figure 3 show that a new resonant point is obtained in the high-frequency band, labeled as *fh1*, demonstrating that this design achieves dual-band operation.

To extend the bandwidth in the low-frequency band, four parasitic strips are introduced into Ant. C, as depicted in Figure 2c. The parasitic strips adopt a rectangular design to ensure better compatibility with the rectangular microstrip patch, thereby maintaining overall structural compactness. The energy is coupled from the microstrip patch to the parasitic strips, thereby generating a new resonant point in the low-frequency band, labeled as *fl2*, as depicted in Figure 3. To further broaden the bandwidth in the high-frequency band, a U-shaped slot is introduced into the microstrip patch of the proposed design, as depicted in Figure 1. The U-shaped slot alters the surface current on the microstrip patch, creating an additional current path. The length of this path is approximately half the guided wavelength at the corresponding frequency. As depicted in Figure 3, a new resonant point, labeled *fh2*, is obtained in the high-frequency band.

In this paper, the proposed design has a total of four controllable resonant points, achieving dual-band broadband characteristics.

## 3. Working Principle

**Figure 4:**
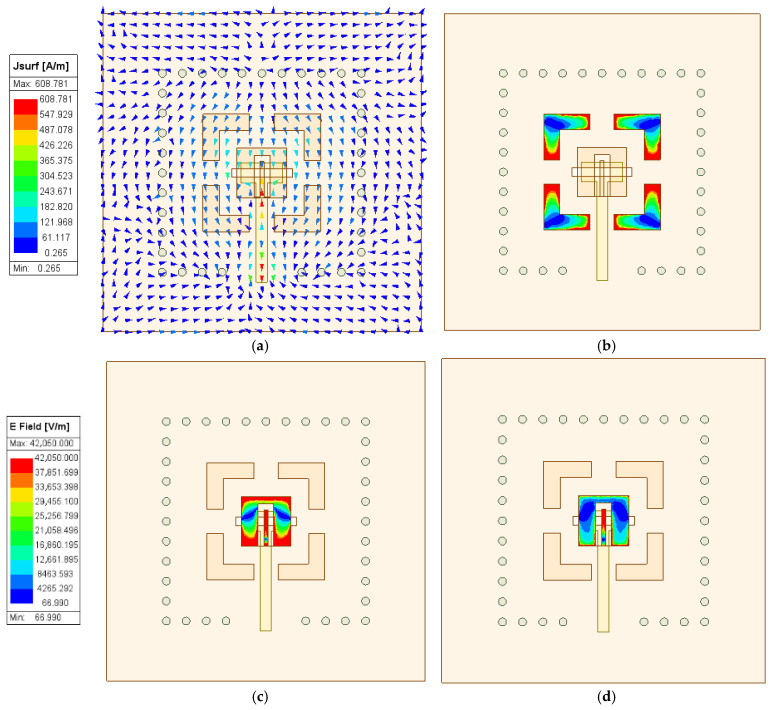
Current and electric field distributions of the designed antenna. (**a**) Current distribution at *fl1*; (**b**) Electric field distribution at *fl2*; (**c**) Electric field distribution at *fh1*; (**d**) Electric field distribution at *fh2*.

At *fl1*, the current distribution of the designed antenna is presented in Figure 4a. As observed, the current is primarily concentrated in the slot antenna. This current distribution indicates that the resonant point is primarily generated by the slot antenna. Based on the above analysis, *fl1* can be optimized using the formula below.

(1) $$fl=\frac{c}{2sl\sqrt{\varepsilon_{r}}}$$

**Figure 5:**
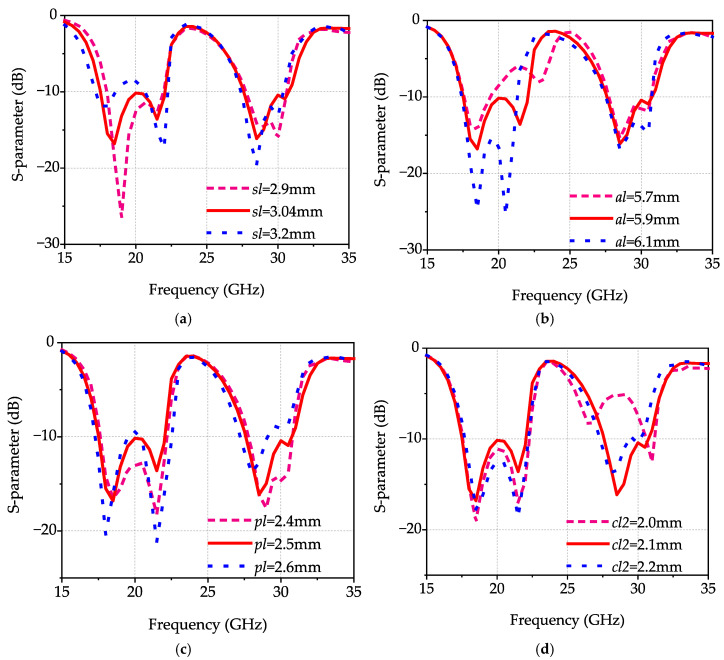
Variation in the simulated reflection coefficient with parameters. (**a**) With different *sl*; (**b**) With different *al*; (**c**) With different *pl*; (**d**) With different *cl2*.

As depicted in Figure 5a, *fl1* can be independently controlled and is determined by the slot antenna dimensions. As *sl* decreases, *fl1* shifts toward higher frequencies, which is consistent with theoretical predictions.

The electric field distribution at *fl2* is depicted in Figure 4b. The electric field energy is primarily concentrated around the four parasitic strips, demonstrating that the resonance at *fl2* is primarily generated by parasitic strips. As shown in Figure 5b, *fl2* can be independently controlled and is determined by the dimensions of the parasitic strips. As *al* decreases, *fl2* shifts toward higher frequencies, which is consistent with the above analysis.

The electric field distributions at *fh1* and *fh2* are presented in Figure 4. The results indicate that the electric fields at *fh1* and *fh2* are primarily distributed on the surface of the microstrip patch, while the electric field at *fh2* exhibits stronger concentration around the U-shaped slot structure. This distribution characteristic indicates that the resonance at *fh1* is primarily generated by the microstrip patch, while the resonance at *fh2* is mainly produced by the U-shaped slot structure. It can be inferred that *fh1* and *fh2* primarily depend on the dimensional parameters of the microstrip patch and the U-shaped slot. The results, as depicted in Figure 5c, indicate that as *pl* decreases, *fh1* shifts toward higher frequencies with a slight perturbation in *fh2*. As illustrated in Figure 5d, with decreasing *cl2*, *fh2* shifts toward higher frequencies while also inducing a slight variation in *fh1.* This effect indicates that co-optimization of the microstrip patch and U-shaped slot dimensions is necessary to achieve excellent dual-band broadband characteristics.

## 4. Antenna Implement

**Figure 6:**
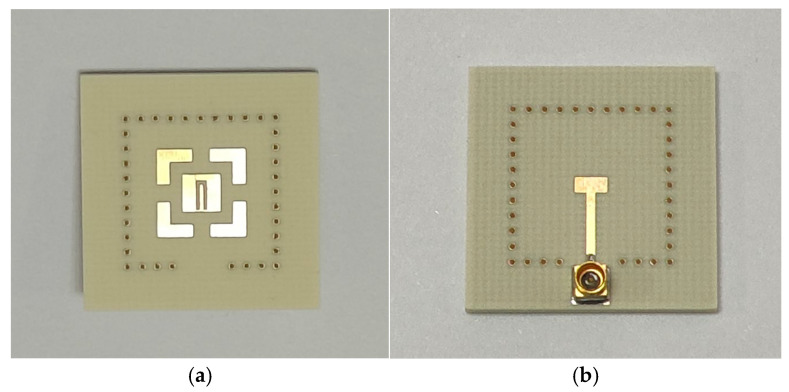
Photograph of the manufactured antenna. (**a**) Top perspective; (**b**) Bottom perspective.

To validate the above theory, the proposed dual-band broadband antenna is designed, manufactured, and experimentally validated. The design is simulated using the High Frequency Structure Simulator (HFSS) and physically tested using an Agilent N5230C network analyzer and Satimo system. Figure 6 presents a photograph of the manufactured antenna.

**Figure 7:**
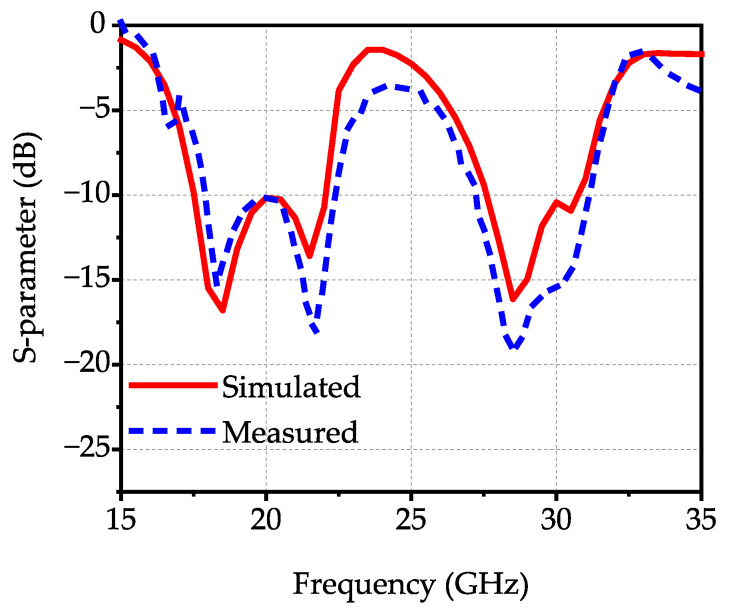
The simulated and experimental S-parameters of the proposed antenna.

**Figure 8:**
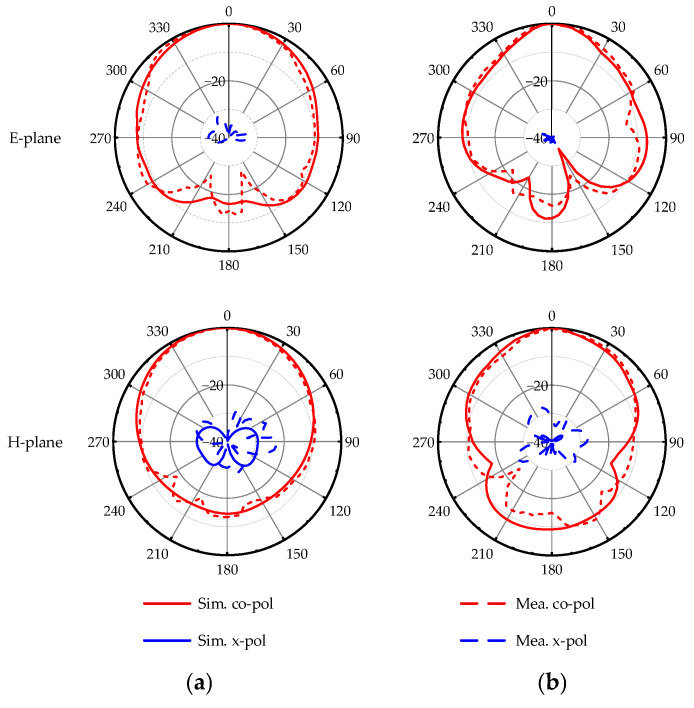
Simulated and experimental normalized radiation patterns. (**a**) At 18.9 GHz; (**b**) At 30.2 GHz.

The proposed antenna’s simulated and experimental results are depicted in Figure 7. The experimental impedance bandwidths (for |S_11_| < −10 dB) are 22.1% (17.9–22.35 GHz) and 13.6% (27.24–31.2 GHz), demonstrating excellent dual-band broadband performance. The normalized radiation patterns are illustrated in Figure 8. The peak gains of the proposed antenna are 6.5 dBi at 18.9 GHz and 4.6 dBi at 30.2 GHz, with a cross-polarization level below –20 dB and high antenna efficiency, demonstrating good radiation performance. In practical fabrication and measurement, the proposed antenna structure not only features a simple manufacturing process but also demonstrates good robustness to manufacturing tolerances, which lays a solid foundation for its subsequent practical applications.

Table 2 provides a comparison between the proposed dual-band antenna and other published designs. Compared with the dual-band designs in [8,20,21,22], the proposed dual-band design has a lower profile as well as wider operating bandwidth. Although the dual-band antennas in [16,25] achieve a lower profile, their dual-band bandwidths are significantly narrower than that of the proposed antenna. Therefore, compared to existing designs, the dual-band antenna proposed in this paper has advantages in terms of bandwidth, profile, radiation performance, and structural complexity.

## 5. Conclusions

A novel dual-band broadband antenna based on structure reuse is designed, fabricated, and measured. The integration of a slot antenna and a microstrip antenna enables dual-band operation. The slot antenna is innovatively designed with a dual function, serving simultaneously as a radiating structure and a feeding structure. This approach enables structure reuse and significantly reduces the structural complexity. To broaden the operating bandwidth, four parasitic strips and a U-shaped slot are introduced into the design. The measurement results show relative bandwidths of 22.1% and 13.6%, with peak gains reaching 6.5 dBi and 4.6 dBi, respectively. The dual-band antenna proposed in this paper demonstrates excellent performance in both bands, with advantages including wide bandwidth, low profile, structural simplicity, and ease of fabrication, which shows its significant potential for dual-band applications.

## Figures and Tables

**Table 1:** Dimensional parameters of the designed structure.

Parameters	Value/mm	Parameters	Value/mm
*ad1*	1.23	*fw2*	1
*ad2*	1.23	*Gl*	16
*al*	5.9	*h1*	1.046
*cl1*	0.8	*h2*	0.254
*cl2*	2.1	*pl*	2.5
*fl*	2.1	*sl*	3.04
*fw1*	0.6	*sw*	0.45

**Table 2:** Comparison of the proposed and other designs.

	Antenna Type	Method	Lower Band BW. (%)	Higer Band BW. (%)	Profile
[7]	Dipole antenna	Shared aperture	7.6	8.69	0.26*λ_L_*
[14]	Microstrip patch	Multiple resonant modes	3.1	9.2	0.05*λ_L_*
[18]	Microstrip patch	Parasitic patch	6.8	11	0.11*λ_L_*
[19]	Magnetoelectric dipole	Magnetic dipoles + electric dipoles	2.19	9	0.15*λ_L_*
[20]	Microstrip patch	Coupled strips	16.7	11.2	0.11*λ_L_*
[25]	Microstrip Patch	Etched slots	4.8	6.9	0.05*λ_L_*
Pro.	Slot antenna	Structure reuse	22.1	13.6	0.08*λ_L_*

*λ_L_* refers to the wavelength of the lower frequency in free space.

## Data Availability

The original contributions presented in this study are included in the article. Further inquiries can be directed to the corresponding author.
